# The Global Occurrences of Cleft Lip and Palate in Pediatric Patients and Their Association with Demographic Factors: A Narrative Review

**DOI:** 10.3390/children11030322

**Published:** 2024-03-08

**Authors:** Farah Asnely Putri, Madhuri Pattamatta, Sheylla Edu September Anita, Tantry Maulina

**Affiliations:** 1 Oral and Maxillofacial Surgery Department, Faculty of Dentistry, Padjadjaran University, Bandung 40132, Indonesia; farah.asnely@fkg.unpad.ac.id; 2 Dentistry Department, Faculty of Medical Sciences, Radboud University, 6525 XZ Nijmegen, The Netherlands; madhuri.pattamatta@radboudumc.nl; 3 Nanjungmekar Community Health Center, Bandung 40394, Indonesia; sheyllaedu@gmail.com

**Keywords:** orofacial cleft, cleft lip, cleft palate, occurrence, prevalence, incidence, demographic, socioeconomic status, educational attainment

## Abstract

Objective: Orofacial clefts are one of the most common abnormalities that occur in the orofacial area. Due to their high prevalence, special attention provided to risk factors and their possible involvement in the occurrence of orofacial clefts is of importance. The objective of this study was to review the current global occurrence of orofacial clefts and the possible linkage of previously investigated risk factors to the occurrence of orofacial clefts. Review: The risk factors of orofacial clefts can be classified into two groups, modifiable risk factors and non-modifiable risk factors. Due to the extent of elaboration of each risk factor in each group, this current narrative review is limited to several mostly investigated risk factors, which included a review of parental age, sexual disparities, educational attainment, and income. Studies indicate that Asians are more likely than other races to have orofacial clefts, with a higher incidence rate in men than in women. There is evidence that the age of the parents is associated with the chance of the occurrence of orofacial cleft. The prevention of orofacial clefts and the distribution of medical resources depend heavily on a thorough understanding of epidemiology on a global scale. Nevertheless, the earlier studies concentrated on more developed nations or areas, and registry data from low-income nations had significant gaps. The findings of this narrative review can be used as the scientific basis for further research within this area. Conclusion: The occurrence rate of orofacial clefts remains high in several regions. Possible associations between parental age, sexual disparities, educational attainment, and family income to the occurrence of orofacial clefts remain contradictory, indicating the importance of further research to obtain more insights.

## 1. Introduction

Orofacial clefts can develop as a syndrome or as a single abnormality, and they are among the most prevalent birth abnormalities worldwide. Orofacial clefts can be classified as congenital cleft lip and/or cleft palate. With regard to their etiology, a combination of external and hereditary variables influences it in most complex instances. Malnourishment, hormone imbalances, drugs, pollutants, and biological variables are considered external factors [1,2,3]. To achieve full closure of an orofacial cleft, promote normal facial development and growth, and enhance the patient’s capacity for social interaction and communication through improved speech, hearing, and facial appearance, the cleft lip and palate repair consists of a complex process that takes a long time, extending from infancy through adolescence and even young adulthood [4,5,6].

Due to the failure of the processes that generate the primary and secondary palates, orofacial cleft has been related to risk factors that women are exposed to during the first three months of pregnancy. These processes involve the lip, alveolar process, hard palate, and soft palate. Cleft lip, cleft palate, and cleft lip and palate might occur due to interferences within this period because the fusion of the upper lip completes during the sixth and eighth week of gestation and the fusion of the hard and soft palates between the eighth and twelfth week. Long-term care is necessary for orofacial cleft-related functional and cosmetic issues. These individuals exhibit a heightened likelihood of morbidity; challenges with nutrition, alterations in speech and hearing, disrupted social interactions, as well as psychological and financial ramifications for their siblings [7].

Due to the complex and long process of recovery, previous studies have revealed orofacial cleft patients’ and families’ quality of life is negatively impacted. Parental stress can be caused by numerous consultations, surgeries, financial consequences, their child’s stigma, and peer victimization. There have also been suggestions of reduced social activity involvement, guilt sentiments, and detrimental consequences on their relationship and job. The caregiver’s quality of life may be negatively impacted by each of these factors [4,8,9]. To view the impact of orofacial clefts from the patient’s perspective, orofacial cleft patients must deal with more than just physical difficulties. Additionally, communication, comprehension, and cognition may be affected by this deformity. They consequently might impact patients’ mental health, well-being, and self-esteem, which in turn affects their social interactions, quality of life, and social life [10,11].

With regard to the occurrence of orofacial clefts in Indonesia, a nationwide prevalence of orofacial clefts in Indonesia increased as much as 0.04% in a five-year period, according to nationwide study performed in Indonesia. The nationwide prevalence of cleft lip in Indonesia is 0.2%, according to the National Guidelines for Medical Services, which deal with the treatment of cleft lip and palate. In Indonesia, 7500 incidences of orofacial clefts are reported every year [12], indicating a quite high prevalence, drawing attention and focus on the continuous investigation within the topic. The objective of this narrative review was to review the current occurrences of orofacial clefts globally, and its association to several risk factors, to provide the most current overview of the orofacial cleft anomaly in children.

## 2. Review

### 2.1. Global Occurrences—Current Number

According to a study conducted by Hlongwa, Levin, and Rispel in 2019 regarding the epidemiology and clinical profile of orofacial clefts patients, it was stated that congenital anomalies caused the death of 303,000 newborn infants worldwide within the period of four weeks after birth. Orofacial clefts were reported as the most common anomaly in the orofacial area, with the occurrence rate of 1 case per 700 live births. In their study, Hlongwa, Levin, and Rispel (2019) also investigated 699 records of children with a median age of 3 years with orofacial clefts in South Africa. Out of the 699 records, it was revealed that 133 children were diagnosed with cleft lip, 247 children were diagnosed with cleft palate, and 305 children were diagnosed with cleft lip and palate [13]. Based on the Global Oral Health Status Report (2022) of the World Health Organization (WHO), it was approximated that oral diseases affect nearly 3.5 billion people worldwide. Orofacial clefts were reported to have a global prevalence of 1 in every 1000–1500 births [14].

In a review study conducted by Salari et al., in 2022, regarding the global prevalence of orofacial clefts involving 57 studies and 17,907,569 individuals, it was revealed that the prevalence of cleft lip per 1000 live birth was 0.3 (95% CI: 0.26–0.34); the prevalence of cleft palate that was based on the results of 59 studies and 21,088,517 individuals per 1000 live birth was 0.33 (95% CI: 0.28–0.38); and the prevalence of cleft lip and palate that was based on 55 studies and 17,894,673 individuals per 1000 live births was 0.45 (95% CI: 0.38–0.52) [1]. To have a more recent overview regarding the occurrence of cleft lip and palate globally, results from the current study that were published between 2018 and 2020 regarding the prevalence of cleft lip and palate in newborns are gathered in Table 1.

In a study performed in Indonesia by Sundoro et al., regarding corrective procedures for children with orofacial clefts between September 2018 and August 2021, it was revealed that 883 children (mean age: 7.94 years old) that were diagnosed with orofacial clefts underwent an operative procedure. Of 883 children, 478 were diagnosed with cleft lip and 299 were diagnosed with cleft palate. From the study, it was also revealed that based on a national survey conducted in Indonesia in 2018, there has been an increase in orofacial cleft prevalence of 0.04% [12]. Another study that was conducted within the Southeast Asian region was a study conducted by Yow, Jin, and Yeo (2021). In their study, Yow, Jin, and Yeo (2021) reported the prevalence of orofacial cleft according to the patient ethnic groups, which was as follows: 17.17 per 10,000 births for Chinese ethnicity, 16.92 per 10,000 births for Malay ethnicity, 10.74 per 10,000 births for Indian ethnicity, and 21.73 per 10,000 births for mixed ethnic origins [26].

Additionally, a study conducted in Colombia by Reina, Brigetty, and Salomon (2023) regarding the occurrence of orofacial clefts in Colombia revealed a total of 15,225 people with orofacial clefts, and the prevalence of cleft lip was 0.93 for every 10,000 births; the prevalence of cleft palate was 1.17 for every 10,000 births; and the prevalence of cleft lip and palate was 1.26 for every 10,000 births (unilateral cleft lip and palate prevalence was 0.83 per 10,000 births, and bilateral cleft lip and palate prevalence was 0.43 per 10,000 births) [27]. Meanwhile, in a review study performed by Shrestha et al. (2023), on the occurrence of orofacial clefts in developing countries, it was revealed that the occurrence of orofacial clefts in Gujrat, India, was 0.73 in 1000 births; the occurrence of orofacial clefts in Nepal was 1.64 per live birth per year; and the occurrence of orofacial clefts in Palestinian territories was 1.01 in 1000 live births [28].

Regardless of the variations in the occurrence rate of orofacial clefts globally, the numbers are generally high, indicating the importance of prevention and risk factor identification. With regard to risk factors, the involvement of several demographic factors has been revealed in previous studies, namely, parental age, sexual disparities, educational attainment, and income. Investigating the relationship between these risk factors and the occurrence of orofacial cleft will provide a solid scientific basis for preventive measures.

### 2.2. Parental Age

Parental age has been proposed as one of the risk factors for orofacial cleft [29,30,31,32]. In a previous study performed by Camilla et al. (2005) regarding the parental age of children with orofacial clefts, it was revealed that as maternal age rises from 20 to 40 years old and paternal age increases from 20 to 50 years old, the occurrence rate of orofacial clefts increases. While precision is reduced by small numbers in these categories, the extreme age groups appear to join this overall pattern to a certain extent. The odds ratio (OR) for cleft lip and cleft lip and palate within the 20–40 age range was 1.20 per 10-year increase in age (95% CI = 1.08–1.33), and for cleft palate exclusively, it was 1.16 per 10-year rise (1.00–1.35). The ORs for fathers between the ages of 20 and 50 were 1.12 (1.02–1.22) for cleft lip and cleft lip and palate and 1.24 (1.10–1.40) for cleft palate [31].

Interestingly, in a previous study performed by de Carvalho et al. (2016) in Brazil, an association between maternal age (for mothers aged 35 years old or less) and the prevalence of orofacial cleft was not found. However, there was an association found between paternal age and the occurrence of orofacial clefts in this study. It was revealed that if the father is less than 40 years old, then there are fewer occurrences of cleft palate compared to cleft lip and palate. Moreover, the association was only found in female children with orofacial cleft but not in male children with orofacial cleft [30].

While in a more recent study conducted by James et al. (2020) regarding parental age and its contribution to the occurrence of orofacial cleft in the Nigerian population, contrary to the finding of Camilla et al. (2005) where the occurrences of orofacial cleft increased with the rise in maternal age, the finding of James et al. (2020) showed that there is a higher percentage of children diagnosed with orofacial cleft if the maternal age is 25 years or less. A significant correlation between the occurrence of orofacial cleft and maternal age was found. When compared to mothers under 25 years old, mothers aged 26–35 showed significantly lower odds of having a child with orofacial cleft (OR: 0.32; 95% CI: 0.16, 0.79; *p*: 0.007). Similarly, mothers over 35 years of age had lower odds of birthing children with orofacial cleft compared to those under 25 (OR: 0.21; 95% CI: 0.06, 0.66; *p*: 0.003). As for paternal age, a less similar manner was found, of which, as the age of the father increases, the probability of occurrence of having children with orofacial cleft is significantly reduced [29].

Additionally, a study conducted by Caramelo et al. (2021) revealed that with an increase in each year of maternal age, the likelihood of birthing a child with orofacial cleft decreases (OR = 0.903). However, no association was found when it comes to paternal age and the occurrences of having a child with orofacial cleft [33]. A study performed by Karina et al. (2020) in Indonesia suggested that the possibility of a mother having a child with orofacial cleft increases if the mother’s age is 35 years and more. Contradictory to this result, no significant association was found between paternal age and the type of orofacial cleft [34].

### 2.3. Sexual Disparities

Sexual difference in the occurrence of orofacial cleft also shows variable results, regardless of the tendency observed in previous studies where male infants are, supposedly, more affected than female infants [26,35,36,37]. In a study performed by Yow et al. (2021), it was revealed that for every 10,000 births, the prevalence of orofacial cleft in male children was 17.72, and the prevalence of orofacial cleft in female children was 15.78. Out of the 363,633 live births during the study period, 115 infants were diagnosed with cleft lip, 249 infants were diagnosed with cleft palate, and 244 infants were diagnosed with cleft lip and palate. From the 115 infants with cleft lip, 72 (62.6%) infants were male, and 43 (37.4%) infants were females; from the 249 infants with cleft palate, 97 (39%) infants were male, and 152 (61%) infants were female; and from 244 infants with orofacial clefts, 143 (58.6%) infants were male, and 101 (41.4%) were female [26].

A previous study conducted by Philipp et al., in 2023, stated that female infants are less affected than male infants when it comes to the occurrence of orofacial cleft. In their study, Philip et al. (2023) examined 404 patients with orofacial cleft. Out of these 404 patients, 58 patients had cleft lip, of which 32 (55.2%) were male, and 26 (44.8%) were female; 140 patients had cleft palate, of which 73 (52.1%) patients were male, and 67 (47.9%) patients were female; and 158 patients had cleft lip and palate, of which 104 (65.8%) patients were male, and 54 (34.2%) were female [35]. From these findings, it can be seen how orofacial clefts are less likely to occur in females than in males, supporting the results reported by Yow et al. (2021). Gender disparities in cleft phenotypes have been suggested to be caused by temporal variations in embryological development. For example, females were more likely to exhibit secondary palate maldevelopment or cleft palate because their palatal development was slower than that of males. One significant risk factor for the incidence of orofacial cleft was, indeed, gender [26].

To further investigate sexual differences in the occurrence of orofacial cleft, a study by Alhayyan et al. (2021) in Saudi Arabia was reviewed. Interestingly, Alhayan et al. concluded that, based on the findings of their study, sexual disparities do not make a significant contribution in the occurrence of orofacial cleft. From their study period, 78 children with orofacial cleft were detected. Out of the 78 children, there were 39 male children with orofacial cleft and 39 female children with orofacial cleft, describing sexual equality instead of sexual differences [36]. Additional information regarding sexual disparities in the occurrence of orofacial cleft was provide by a study performed by Impellizzeri et al. (2019), where it was stated that cleft lip is more predominant in females, showing a ratio of 0.8:1 (male/female), while cleft lip and cleft lip and palate are more predominant in males, with a ratio of 1.5:1 (male/female) [37].

There have been some proposed mechanisms regarding the sexual difference in the occurrence of orofacial cleft. Although a common explanation for these sexual difference variations has not yet been established, it might be due to the difference in the development of important phases of the craniofacial structure of male and female embryos. But when it comes to the general categories of orofacial cleft and the orofacial cleft sub-phenotypes in particular, there has not been much discussion on gender disparities in the timing of these crucial developmental stages in the literature up until now. It is important to note that only fusion faults of the primary palate develop in the earlier period, whereas those of the secondary palate arise in the later period. Only in the later stage, primary and secondary palate differentiation abnormalities arise. The lip, or premaxilla/maxilla, or alveolus, extending to the incisive foramen, is a part of the primary palate. The secondary palate is made up of the uvula, hard palate, and soft palate. Therefore, the difference in timing of how female and male embryos develop may contribute towards the occurrence of cleft lip and palate [38]. Additionally, Carson et al. (2018) stated how genetic variation may also play a role [39].

### 2.4. Parental Educational Attainment

Another demographic characteristic that is thought to contribute to the occurrence of orofacial cleft is parental educational level [7,40,41]. Parental educational attainment is considered a modifiable risk factor. In a systematic review conducted by Ichingolo et al. (2022) regarding modifiable risk factors for non-syndromic orofacial clefts, low paternal educational level is known to be a contributing factor towards the occurrence of orofacial clefts [41]. Additionally, a study conducted by Figuireido et al. (2015) also highlighted the possible correlation between lower maternal and paternal education levels and the occurrence of orofacial cleft [42]. In a recent study conducted by da Silva et al. (2023), it was shown that the maternal education level of children that were born with an orofacial cleft is average [40].

In a study performed by Regina Altoé et al. (2020) regarding the impact of parental exposure on orofacial cleft’s occurrences, it was indicated that a correlation (*p* = 0.007) between the incidence of orofacial cleft and low paternal education was found. Although there is a direct correlation between education and income, a poor level of paternal education may have prevented the expectant mother from receiving enough nourishment, which could have led to the development of congenital deformity [7]. It has been acknowledged in previous studies that poor educational achievement can have a long-term negative effect on outcomes related to employment, social interactions, mental health, and physical health [43], including a possible effect on the occurrence of orofacial cleft.

To further investigate regarding the possible association between parental educational attainment and the occurrence of orofacial pain, a study performed by Ly et al. (2017) provided a contradictory finding compared to previous studies mentioned above. Ly et al. (2017) indicated that there is no association between paternal level of education and the occurrence of orofacial cleft [44]. To add a variation in findings, a study performed by Vu et al. (2022) suggested that the level of education impacts the occurrence of orofacial clefts, where having a bachelor’s degree or higher in maternal education was linked to a lower incidence of both cleft lip and cleft palate (OR = 0.73; 95 percent CI, 0.63, 0.85; *p* < 0.001), but not cleft palate alone (OR = 0.87; 95 percent CI, 0.68, 1.11; *p* = 0.257) [45].

Considering that educational attainment is known as one of health’s social determinants, it is important to note how previous studies have linked low educational attainment to the occurrence of diseases [46,47]. Regardless of the acknowledged association between educational attainment and the occurrence of orofacial clefts, the contradiction or variation between research findings does not yet allow a solid conclusion.

### 2.5. Income

Socioeconomic status, including income, has been proposed as one of the risk factors of orofacial cleft. It was proposed that orofacial cleft is more common to occur in children that come from a lower socioeconomic status [45,48]. A connection between a lower socioeconomic status and a higher incidence of orofacial clefts has also been suggested by recent studies [49,50]. The evidence is contradictory, nevertheless, as other studies have found no link between the likelihood of orofacial clefts and a lower socioeconomic class. The low number of previous studies, their small coverage of research area, and the existence of confounders all further reduce the strength of the evidence. Understanding the link between socioeconomic position and the occurrence of orofacial clefts is crucial since a cleft diagnosis may result in diminished employability and prolong the poverty cycle if left untreated [45].

In a study conducted by Wang et al. (2023) regarding the global, regional, and national burden of orofacial clefts, the high occurrence of orofacial cleft in Norway and Finland is interestingly not associated with a low socioeconomic status as both countries have excellent medical care and considered as high-income countries [48]. A rather different finding was shown by a study conducted by Sabbagh et al. (2023) where the difference in monthly family income is reflected in the occurrence of orofacial clefts, and where those with a higher family income showed a lower occurrence of orofacial clefts [51].

Different socioeconomic status levels have been correlated with different types of orofacial clefts; for example, cleft lip has been reported to be more common in a population with a lower socioeconomic status [52]. According to two studies [52,53], there is a correlation between the incidence of a cleft lip and the absence of prenatal folic acid supplementation. Consequently, there may be a connection between cleft type, prenatal folic acid consumption, and socioeconomic status. Further research is necessary since a large number of moms in low- and middle-income countries do not have easy access to folic acid supplements or proper prenatal care [54].

Due to the limited availability of nutrient-dense food, being poor has been linked to nutritional deficits [55]. Rural residents have the danger of coming into contact with heavy metals through tainted food or contaminated water, which has been connected to cleft lip and palate [42,56]. Due to the limited access to healthcare, mothers with a low socioeconomic status are less likely to obtain advice regarding healthy eating during pregnancy [42,52,56,57]. A low socioeconomic status has an even greater detrimental effect on the standard of education that an individual’s experiences, which ultimately leads to the lack of knowledge as well as awareness regarding environmental risk factors that may result in negative pregnancy outcomes [54].

## 3. Conclusions

Children and their families are affected by orofacial clefts in several ways, including social, psychological, and economic aspects. This narrative review highlighted the possible association between the several demographic factors and the occurrence of orofacial cleft. Based on the findings of this narrative literature review, the different aspects revealed were as follows: (1) the current global occurrence rate of orofacial clefts varies according to geographical location and/or race; (2) regardless of its contribution to the occurrence of orofacial cleft, parental age showed a high variation; (3) in general, orofacial cleft is more predominant in male children compared to female children; (4) lower educational attainment is almost always associated with the high occurrences of orofacial cleft; and (5) the high occurrence of orofacial cleft is associated with lower income. However, considering the nature of this study, a more systematic literature review where the risk of bias of each study is validated should be conducted, as it will provide robust evidence.

## 4. Future Direction

Regularly conducted, standardized studies investigating the involvement of modifiable and non-modifiable risk factors in the future have the potential to provide a thorough overview of the global occurrence of orofacial clefts as well as an overview of their yearly patterns.

## Figures and Tables

**Table 1:** The prevalence of cleft lip (CL), cleft palate (CP), and cleft lip and palate (CL&P) in newborns from global studies published between 2018 and 2022 (adjusted from Salari et al., 2022) [1].

Study by	Year	Location	Total Samples	Number of CL	Number of CP	Number of CL&P
Andrew, T. [15]	2018	California	1,458,856	-	922	-
Corona-Rivera, J. R. [16]	2018	Mexico	81,193	30	51	146
Paaske, E.B. [17]	2018	Denmark	182,907		127	
Wang, M. [18]	2018	China	347,137	295	37	118
Yang, Y. [19]	2018	China	50,234	31	17	47
Alswairki, H. J. R. [20]	2019	Egypt	237,783	23	20	44
Imay, Y. [21]	2019	Japan	97,902	-	51	-
Luo, Y. L. [22]	2019	China	597,306	-	177	-
Mezawa, H. [23]	2019	Japan	101,825	125	83	208
Bronberg, R. [24]	2021	Argentina	228,208	54	60	220
Zhou, Y. [25]	2022	China	238,712	1759	3016	3561

## Data Availability

All data presented in this review can be found within the references list.
